# Sir Erastmus Wilson

**Published:** 1882-02

**Authors:** 


### SIR ERASMUS WILSON.

The Prime Minister has communicated to Mr. Erasmus Wilson the intention of the Crown to confer a knighthood on him, in recognition of his professional position, and in consideration of his munificent gifts for the support of hospitals and the encouragement of medical study. Sir Erasmus Wilson occupies this year the distinguished position of President of the Royal College of Surgeons of England, and has long been at the head of the dermatologists of England, and foremost in Europe in that specialty. Among his munificent gifts to hospitals has been the erection, at his sole cost, of a wing of Margate Infirmary, involving an outlay of £30.000, in addition to liberal donations to the Royal Medical Benevolent College at Epsom. He has contributed to the encouragement of medical study by the endowment of a chair at the College of Surgeons, and the provision of a collection of objects suitable for instruction. It would be difficult to use more worthily, in the evening of a successful professional life, the surplus of the means which have been accumulated ; and every one will feel that the honor of knighthood is not at all in excess of the claims of the dignity of the position to which Mr. Wilson had attained, and the munificence which has distinguished him.

